# Scaffold attachment factor B1 (SAFB1) heterozygosity does not influence Wnt-1 or DMBA-induced tumorigenesis

**DOI:** 10.1186/1476-4598-8-15

**Published:** 2009-03-06

**Authors:** Benny Abraham Kaipparettu, Klaudia M Dobrzycka, Ora Britton, Adrian V Lee, Alan J Herron, Yi Li, Michael T Lewis, Daniel Medina, Steffi Oesterreich

**Affiliations:** 1 Lester and Sue Smith Breast Center, Departments of Medicine, Molecular and Cellular Biology, Baylor College of Medicine, Houston, TX, USA; 2 School of Medicine and Dentistry, College of Life Sciences and Medicine, University of Aberdeen, Aberdeen, UK; 3 Center for Comparative Medicine and Department of Pathology, Baylor College of Medicine, Houston, TX, USA

## Abstract

**Background:**

Scaffold Attachment Factor B1 (SAFB1) is a multifunctional protein which has been implicated in breast cancer previously. We recently generated SAFB1 knockout mice (SAFB1^-/-^), but pleiotropic phenotypes including high lethality, dwarfism associated with low IGF-I levels, and infertility and subfertility in male and female mice, respectively, do not allow for straightforward tumorigenesis studies in these mice. Therefore, we asked whether SAFB1 heterozygosity would influence tumor development and progression in MMTV-Wnt-1 oncomice or DMBA induced tumorigenicity, in a manner consistent with haploinsufficiency of the remaining allele.

**Methods:**

We crossed female SAFB1^+/- ^(C57B6/129) mice with male MMTV-Wnt-1 (C57B6/SJL) mice to obtain SAFB1^+/+^/Wnt-1, SAFB1^+/-^/Wnt-1, and SAFB1^+/- ^mice. For the chemical induced tumorigenesis study we treated 8 weeks old SAFB1^+/- ^and SAFB^+/+ ^BALB/c mice with 1 mg DMBA once per week for 6 weeks. Animals were monitored for tumor incidence and tumor growth. Tumors were characterized by performing H&E, and by staining for markers of proliferation and apoptosis.

**Results:**

We did not detect significant differences in tumor incidence and growth between SAFB1^+/+^/Wnt-1 and SAFB1^+/-^/Wnt-1 mice, and between DMBA-treated SAFB1^+/+ ^and SAFB1^+/-^mice. Histological evaluation of tumors showed that SAFB1 heterozygosity did not lead to changes in proliferation or apoptosis. There were, however, significant differences in the distribution of tumor histologies with an increase in papillary and cribriform tumors, and a decrease in squamous tumors in the SAFB1^+/-^/Wnt-1 compared to the SAFB1^+/+^/Wnt-1 tumors. Of note, DMBA treatment resulted in shortened survival of SAFB1^+/- ^mice compared to their wildtype littermates, however this trend did not reach statistical significance.

**Conclusion:**

Our data show that SAFB1 heterozygosity does not influence Wnt-1 or DMBA-induced mammary tumorigenesis.

## Background

Scaffold Attachment Factor B1 (SAFB1) is a multifunctional protein that is involved in RNA processing, transcriptional regulation, and stress response [1]. SAFB1 has previously been implicated in breast tumorigenesis. SAFB1 functions as an estrogen receptor α (ERα) corepressor [2], and its overexpression in cultured cells leads to growth inhibition. Importantly, SAFB1 mutations have been identified in microdissected breast tumors but not in the normal adjacent tissue, and SAFB1's chromosomal locus displays extremely high loss of heterozygocity LOH (78%) in invasive breast cancer [3]. There is no significant different in LOH frequencies between ER-positive and ER-negative tumors, and other *in vitro *observations [4] suggest that SAFB1 could exert its effect on tumorigenesis, at least in part, through ER-independent mechanisms. This is further supported by our recent observation that loss of SAFB1/SAFB1 is associated with worse overall survival of breast cancer patients, but does affect Tamoxifen response [5]. Collectively, these data suggest that SAFB1 loss affects breast tumorigenesis in ER-dependent and independent manner.

We have generated SAFB1 knockout (SAFB1^-/-^) mice [6] which are characterized by high prenatal and neonatal lethality, growth retardation associated with low serum insulin-like growth factor I (IGF-I) levels, as well as female subfertility and male infertility. The SAFB1^-/- ^mice that are born alive succumb to frailty, often associated with infections, and die of unknown reasons at an early age. These pleiotropic phenotypes of SAFB1^-/- ^mice make tumorigenesis studies difficult to perform, and we therefore asked whether partial SAFB1 loss in SAFB1^+/- ^mice would influence mammary tumor formation or progression. Therefore, we used two different tumor models; transgenic MMTV-Wnt-1 oncomice, and a chemical carcinogen model, in which tumors were induced with 7, 12-dimethylbenz (a) anthracene (DMBA).

Genes of the Wnt family function in the regulation of growth and differentiation of the mammary gland [7]. Mutation or deregulation of several components and transcriptional targets of the Wnt signaling have been observed in human cancers, including breast [8-12]. Mammary tumors induced by mouse mammary tumor virus (MMTV) infection have revealed oncogenes involved in murine mammary tumorigenesis [13], and Wnt-1 was the first proto-oncogene discovered as a gene frequently activated in mammary tumors arising in mice infected with MMTV [14]. About 50% of MMTV-Wnt-1 transgenic female mice develop mammary tumors by 5–6 months of age [15], and these mice have therefore frequently been used to study the effect of potential tumor suppressor or oncogenes on mammary gland tumorigenesis (for review, see [16]).

DMBA is a prototypical polycyclic aromatic hydrocarbon (PAHs) with carcinogenic and immunosuppressive effects in various species [17]. Since it was first reported by Huggins *et al *[18], DMBA-induced mammary tumors have been widely used as a spontaneous cancer model in laboratory animals including rats [19] and mice [20].

In this study, we have analyzed the effect of partial SAFB1 loss on Wnt-1 and DMBA-induced tumorigenesis. Here show that SAFB1 heterozygosity does not affect tumor incidence and tumor growth. However, we observed changes in the Wnt-1 tumor histopathology, and a trend towards shorter survival in DMBA-treated SAFB1^+/- ^mice.

## Results

### SAFB1 protein expression is decreased in SAFB1^+/- ^mice

**Figure 1:**
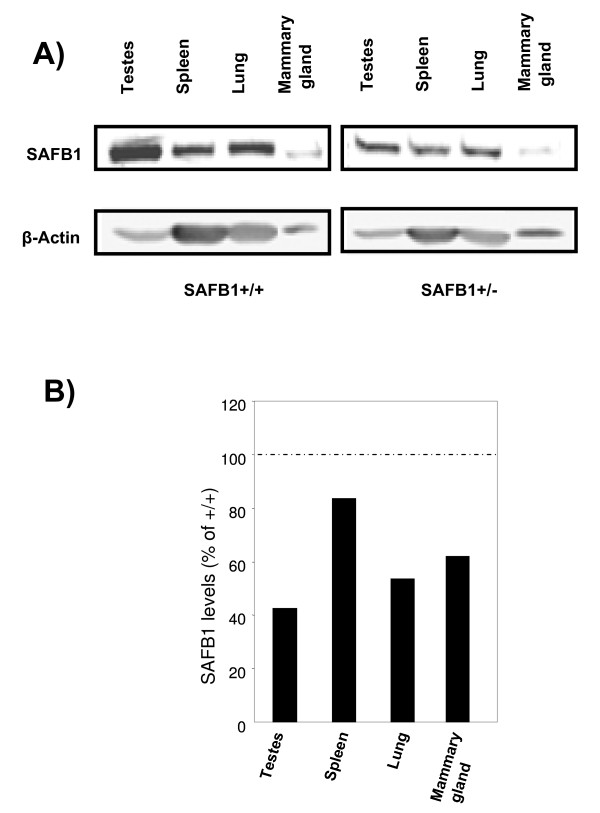
**SAFB1 protein expression in tissues from SAFB1^+/+ ^and SAFB1^+/- ^mice**. A) A representative immunoblot using lysates from different tissues, and antibodies, as indicated. β-actin was used as a loading control. Blots are shown are from mice on a C57Bl/6J × 129/Sv genetic background, but is also representative of difference observed in mice on a BALB/c background. B) Percentage of SAFB1 protein levels in SAFB1^+/- ^tissues, corrected for b-actin levels, relative to protein levels in SAFB1^+/+ ^mice.

First, we set out to confirm decreased SAFB1 protein levels in SAFB1^+/- ^mice using a number of different tissues, including testes, spleen, lung, and mammary gland (Figure 1A). Quantification revealed an average decrease of 42% in the SAFB1^+/- ^mice, compared to SAFB1^+/+ ^mice (Figure 1B). A similar decrease was observed in the tumors, where we detected an average of 53% decrease of SAFB1 protein expression in SAFB1^+/-^/Wnt-1 tumors compared to SAFB1^+/+^/Wnt-1 tumors (data not shown).

### Tumor-free survival and tumor growth

#### SAFB1-Wnt-1 mice

**Figure 2:**
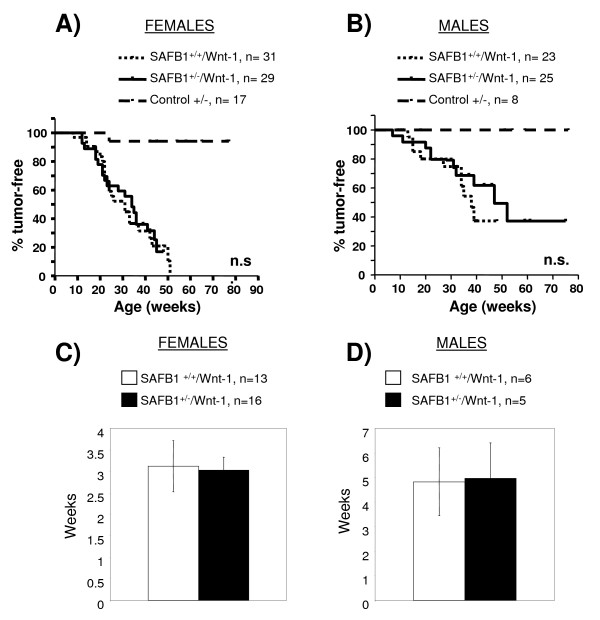
**Effect of SAFB1 heterozygosity on Wnt-1-induced tumorigenesis**. Kaplan-Meier survival curves of female (A) and male (B) SAFB1^+/+^/Wnt-1 and SAFB1^+/-^/Wnt-1 mice. Ages in weeks were recorded at the time of first tumor observation. C) and D) Growth rates of tumors in female and male Wnt-1 transgenic mice, respectively, on a SAFB1^+/+ ^or SAFB1^+/- ^background. The graphs represent time in weeks for tumor growth from detection to 1000 mm^3^, with error bars representing SEM.

We crossed female SAFB1^+/- ^mice with male MMTV-Wnt-1 transgenic mice to obtain experimental mice. There was no significant difference in time to tumor formation between SAFB1^+/+^/Wnt-1 and SAFB1^+/-^/Wnt-1 in both females (Figure 2A) and males (Figure 2B). The median time to tumor formation (MTTF) was 31 weeks (n = 31) for female SAFB1^+/+^/Wnt-1, and 34 weeks for female SAFB1^+/-^/Wnt-1 mice (n = 29). Similarly, we did not detect a significant difference in MTTF between SAFB1^+/+^/Wnt-1 (n = 23) and SAFB1^+/-^/Wnt-1 (n = 25) male mice. Thus, partial SAFB1 loss does not accelerate the formation, or alter the incidence of Wnt-1 induced mammary tumors.

Since tumor incidence was not significantly affected by partial SAFB1 loss, we asked whether loss of one SAFB1 allele would result in accelerated rates of tumor growth, as has been described for other models such as p21^+/-^/Wnt-1 [21]. We therefore analyzed length of time necessary for tumors to grow from < 50 mm^3 ^(time of detection by palpation) to 1000 mm^3^. Tumors in female mice grew slightly faster (Figure 2C) than in male mice (Figure 2D), however, there was no significant difference between SAFB1^+/+^/Wnt-1 and SAFB1^+/-^/Wnt-1 animals in either group.

Since Wnt-1 mice can develop lung metastases [22], we also asked whether partial loss of SAFB1 would affect the metastatic potential in our animals. We microscopically analyzed H&E-stained lung tissue, and detected metastases in 4 out of 13 SAFB1^+/+^/Wnt-1, and in 3 out of 8 SAFB1^+/-^/Wnt-1 mice which was not statistically different. Thus, SAFB1 heterozygosity did not affect the development of lung metastases in the Wnt-1 mice.

IGF-I plays an important role in cell proliferation, differentiation, and apoptosis, and it has also been linked to malignant transformation and breast cancer pathogenesis [23-26]. Numerous studies have shown that a reduction of circulating IGF-I is associated with a decrease in carcinogen and oncogenes induced cancer (in particular, breast and colon cancer) [27]. Interestingly, SAFB1^-/- ^mice are characterized by growth retardation, which was correlated with low serum IGF-I levels [6]. However, we did not detect a significant difference in IGF-I levels between SAFB1^+/+^/Wnt-1 or SAFB1^+/-^/Wnt-1 mice (data not shown), and can thus exclude the possibility that decreased IGF-I levels could mask an effect of SAFB1 heterozygosity on tumorigenesis.

#### DMBA treatment

We next analyzed the effect of SAFB1 heterozygosity on DMBA induced tumorigenesis in BALB/c mice. This study comprised of 67 female mice (34 SAFB1^+/+ ^and 33 SAFB1^+/-^) in the treatment group, and 15 mice (8 SAFB1^+/+ ^and 7 SAFB1^+/-^) in the vehicle treated control group.

**Figure 3:**
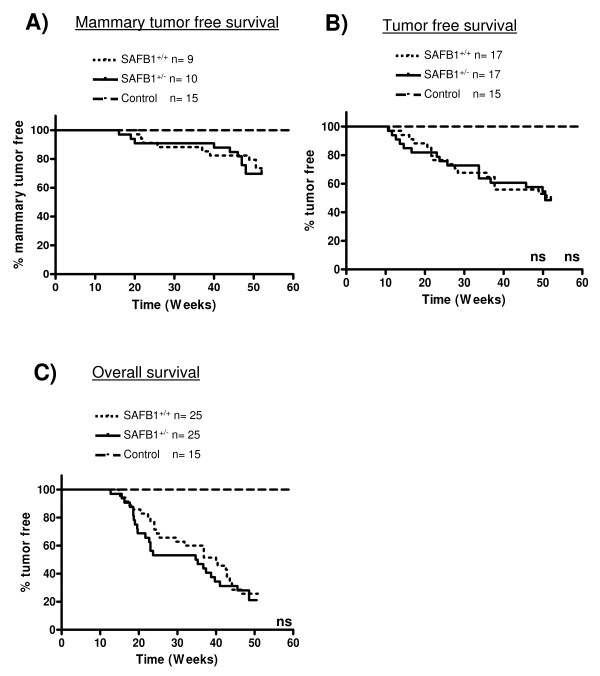
**Effect of SAFB1 heterozygosity on DMBA induced tumorigenesis**. Kaplan-Meier survival curves of mammary gland tumor free (A), all tumor free (B), and overall survival (C) in DMBA-treated SAFB1^+/+ ^and SAFB1^+/- ^mice. Time in weeks were recorded from the first day of DMBA treatment to the time of first tumor observation (A, B), and death or sacrifice of animals due to comorbidity (C).

We first analysed the development of mammary tumors after DMBA treatment. Seventeen mice (28.4%) of the DMBA treated animals developed mammary tumors, however there was no significant difference in tumor development between SAFB1^+/+ ^(n = 9) and SAFB1^+/- ^(n = 10) mice (Figure 3A).

In addition to the mammary gland tumors, animals also developed tumors in other organs including skin, blood, intestine, liver, and lung. A total of 34 animals (50.7%) developed at least one tumor during the study period (17 SAFB1^+/+ ^and 17 SAFB1^+/-^), but again there was no significant difference in tumor free survival (from all tumors) between SAFB1^+/+ ^and SAFB1^+/- ^mice (Figure 3B).

Next we analyzed the overall survival (OS) of the DMBA treated animals. The data captured for OS included death as a result of tumorigenesis, and sacrifice due to co-morbidity of unknown reasons. Interestingly, there was a trend for shorter OS in the SAFB1^+/- ^mice; the median survival was 40 weeks for SAFB1^+/+^, and 35 weeks for SAFB1^+/- ^mice (Figure 3C). To investigate whether this could be due to differential effects of DMBA on the hematopoietic system, we performed complete blood count (CBC) and blood smear test for 15 DMBA treated mice (7 SAFB1^+/+ ^and 8 SAFB1^+/-^), and 2 control mice. Although one SAFB1^+/- ^mice developed B-cell lymphoma, all other blood parameters including red blood cell count, white blood cell count, platelet count, amount of hemoglobin, mean corpuscular volume, mean corpuscular hemoglobin, and mean corpuscular hemoglobin concentration, were not significantly different between both genotypes (data not shown), suggesting that the observed trend was unlikely due to a defect in hematopoietic system in the SAFB1^+/- ^mice. In summary, SAFB1 heterozygosity did not lead to statistically significant effects on DMBA-induced tumorigenesis.

### Histological analysis of tumors

#### SAFB1-Wnt-1 tumor

**Figure 4:**
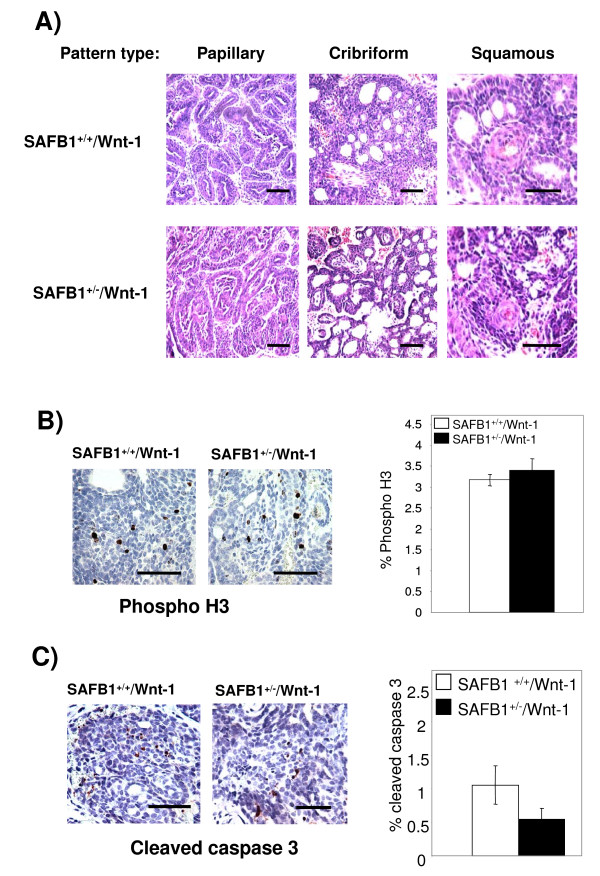
**Histological evaluation of Wnt-1 tumors of different SAFB1 genotype**. A) Both SAFB1^+/+^/Wnt-1 and SAFB1^+/-^/Wnt-1 mice exhibit papillary, cribriform and squamous tumor type patterns. Representative pictures are shown (magnification 20×). B) Tumors found in the female (n = 10 per each genotype) mice were sectioned and stained with phospho H3 antibody. Representative pictures are shown (magnification 40×). The graph represents the quantification of phospho H3-positive cells/field. Error bars represent SEM. C). Wnt-1 tumours found in the female mice were sectioned and stained with caspase 3 antibody (n = 10 per each genotype). Representative pictures are shown (magnification 40×). The graph shows quantification of caspase 3-positive cells/field, with error bars representing SEM (p = 0.0742). Bars represent 100 μm.

We next asked whether partial SAFB1 loss would lead to changes in the histopathology of the Wnt-1 tumors. H&E staining revealed that tumors of both SAFB1 genotypes were well-differentiated, and mainly characterized by three tumor patterns: papillary, cribriform, and squamous (Figure 4A). There was a small difference in the pattern distribution between the two genotypes: while the papillary, cribriform, and squamous pattern represented 45–50%, 40–45%, and <5% distribution respectively, in SAFB1^+/+^/Wnt-1 tumors, they represented 70%, 25% and <5% distribution, respectively, in SAFB1^+/-^/Wnt-1 tumors (p = 0.01).

Next, we set out to determine whether there was a difference in proliferation and apoptosis between SAFB1^+/+^/Wnt-1 and SAFB1^+/-^/Wnt-1 tumors. Therefore, we stained the tumors using BrdU antibody to detect the percentage of cells in S phase, and using phosphorylated H3 (phospho H3) antibody to detect mitotic cells. We did not observe significant changes in the mitotic figures (Figure 4B), and in number of BrdU-positive cells between SAFB1^+/+^/Wnt-1 and SAFB1^+/-^/Wnt-1 mice (data not shown). Similarly, we did not detect a significant difference in rates of apoptosis, as measured by IHC using antibodies for cleaved caspase 3, although there was a trend towards decreased apoptosis in the SAFB1^+/-^/Wnt-1 mice (p = 0.0742 as calculated by unpaired t test, n = 10 per each genotype) (Figure 4C). Together, these data show that partial SAFB1 loss does not affect proliferative and apoptotic rates in Wnt-1 tumors.

#### DMBA induced tumors

**Figure 5:**
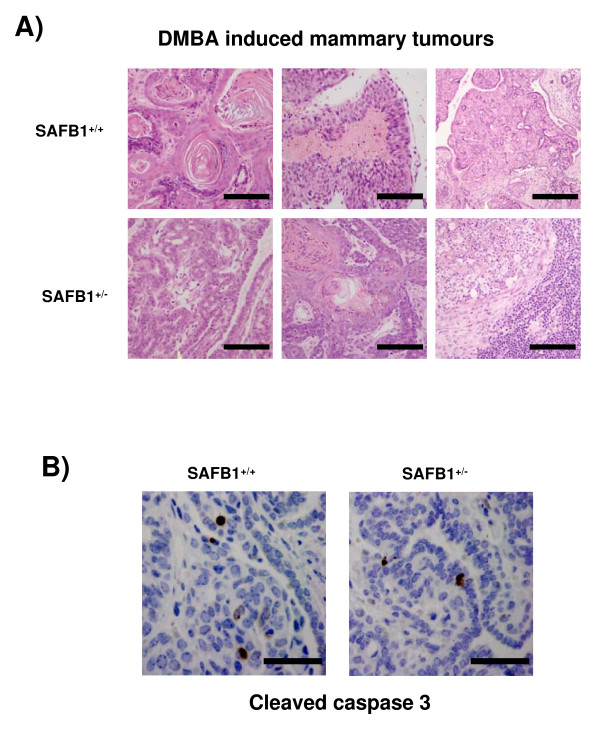
**Histological evaluation of DMBA induced tumors of different SAFB1 genotype**. A) Both SAFB1^+/+ ^and SAFB1^+/- ^mice mostly exhibit squamous tumor type patterns. Representative pictures are shown (magnification 20×). B) DMBA-induced tumors were stained with cleaved caspase 3 antibody. Representative pictures are shown (magnification 40×). Bars represent 100 μm for A and 50 μm for B.

Finally, we determined whether SAFB1 heterozygosity would lead to changes in the histopathology of the DMBA-induced mammary tumors. H&E staining revealed similar histopathology in both groups, in that the tumors mostly contained keratinized squamous epithelial cells (Figure 5A). We also did not detect any significant difference in the percentage of BrdU-positive cells (data not shown), and cleaved caspase-3 positive cells (Figure 5B) between the two SAFB1 genotypes. We thus conclude that SAFB1 heterozygosity did not affect proliferative and apoptotic rates in DMBA-induced tumors.

## Discussion

To better understand SAFB1's role in tumorigenesis *in vivo*, we have analyzed the effect of partial SAFB1 loss on Wnt-1 and DMBA induced tumorigenesis. Our results show that SAFB1 heterozygosity does not accelerate incidence or growth of either Wnt-1 or DMBA-induced tumors.

The absence of an effect could suggest that SAFB1 heterozygosity is not sufficient to cause an increase in tumorigenesis. Indeed, the classical Knudson's two-hit hypothesis states that tumor suppressor genes are recessive, and that both alleles must be inactivated for tumorigenesis [28]. This model was based on the repeated observation that familial cancers often result from the initial germline mutation of one allele of a tumor suppressor gene, followed by subsequent somatic mutation or loss of the second allele. Consistent with this, there are numerous studies showing that loss of a single allele of a tumor suppressor gene does not increase tumorigenesis. For example, partial p53 loss did not accelerate Wnt-1 [29] or DMBA-induced mammary tumorigenesis [30]. Similarly, Brca1 and Brca2 heterozygosity did not affect tumor formation induced by the Wnt-1 transgene [16], and partial deletion of the INK4a locus that encodes both p16^INK4a ^and p19^ARF ^did not result in increased DMBA-induced tumor formation [31]. However, there are also many reports where heterozygosity results in significantly increased tumorigenicity, thus demonstrating haploinsufficiency of the remaining allele. For example, inactivation of a single allele of the gene encoding the cyclin-dependent kinase inhibitor p27^Kip1 ^was sufficient to sensitize mice to radiation and carcinogen-induced tumorigenesis [32]. Dmp1-heterozygote mice treated with DMBA or ionizing radiation accelerated tumorigenesis compared to the wild type [33]. Similarly, MMTV-Wnt-1 transgenic mice which are heterozygous for PTEN developed mammary tumors earlier than the PTEN wild type mice [22]. At this point of time, we cannot exclude the possibility that partial SAFB1 loss would influence the tumorigenicity in other tumor models. For example, as discussed above, partial p53 loss did not cooperate with Wnt-1 [29], however, it did accelerate tumor development in MMTV-Ras [34] and MMTV-Myc [35] mice models.

Our study specifically addressed the question of the effect of SAFB1 heterozygosity in hormone-independent mammary gland tumors. Both, *in vitro *[4,36] and *in vivo *[5] data suggest that SAFB1 has ER-independent activities. Unpublished studies from our group showed that SAFB1 heterozygosity results in increased ER activity in MEFs, both in the absence and presence of ligand, and thus future studies should address potential effects of SAFB1 heterozygosity in development of hormone-dependent tumors, such as the p53^-/- ^tumors [37].

Two interesting observations from this study warrant further discussion. First, we observed a non-significant trend towards a shorter overall survival of SAFB1^+/- ^mice compared to the SAFB1^+/+ ^mice after DMBA treatment. We did not find any obvious defects in the haematopoietic system but the increase in lethality could be due to increased tumor development in other organs, or defects in the immune system, resulting from the partial loss of SAFB1. Second, we observed subtle differences in the histological distribution pattern of the papillary and cribriform tumor types between SAFB1^+/+^/Wnt-1 and SAFB1^+/-^/Wnt-1 tumors. The Wnt pathway is known to influence cell fate determination, stem cells renewal, and expansion of progenitor cells in a number of tissues, including the mammary gland [38-40]. Wnt1-ransformed progenitor cells might differentiate into multiple cells types, resulting in tumor heterogeneity. A role of SAFB1 affects in this function of Wnt-1 could explain the altered histology of tumors in SAFB1^+/-^/Wnt-1 tumors.

In summary, we have shown that partial SAFB1 loss does not cooperate with Wnt-1 or DMBA induced tumorigenesis. Future studies using conditional deletion of both alleles will address the question of whether complete loss of SAFB1 expression results in increased mammary gland tumorigenesis.

## Methods

### Animal models

MMTV-Wnt-1 transgenic mice (a kind gift from Dr. Larry Donehower, Baylor College of Medicine) and those that carry a mutant allele of SAFB1 have been described previously [6,15]. Male Wnt-1 mice (on a mixed SJL and C57BL/6 background) were crossed with SAFB1 heterozygous females (on a mixed C57BL/6J and 129Sv background) to generate the three genotypes used in our study: SAFB1^+/+^/Wnt-1, SAFB1^+/-^/Wnt-1, and SAFB1^+/-^. For DMBA analysis we used mice on a BALB/c background. All animals were maintained in a pathogen-free facility at Baylor College of Medicine, and fed a diet of standard food and water *ad libitum*. All procedures were approved by the BCM Institutional Animal Care and Use Committee.

### Genotyping and Immunoblotting

The genotypes were determined by isolating tail DNA and performing PCR. Primers used to detect the intact SAFB1 gene (5'-TAT TAT CAT CTG CAT TCA CCA GTT G-3' and 5'-TCT GTG CTC CCA TCA TCT GTC TTC TTG-3') generate a 715 bp PCR product. Primers used to detect LacZ (5'-CTG CTG ATG AAG CAG AAC AAC TTT A-3' and 5'-GAC AGA TTT GAT CCA GCG ATA CAG-3') generate a PCR product of 306 bp. Primers used to detect Wnt-1 transgene (5'-GAA CTT GCT TCT CTT CTC ATA GCC-3' and 5'-CCA CAC AGG CAT AGA GTG TCT GC-3') generate a 350 bp PCR product.

To confirm decreased SAFB1 protein levels of heterozygous mice compared to wild type mice, snap-frozen testes, spleen, lung, kidney, uterus and mammary gland tissues from SAFB1^+/+^and SAFB1^+/- ^mice were crushed, lysed in 5% sodium dodecyl sulfate (SDS), and sonicated. Proteins were resolved by SDS polyacrylamide gel electrophoresis and transferred to a nitrocellulose membrane. After blocking (PBS and 0.1% Tween 20 (PBST) + 5% milk), membranes were probed with SAFB1 [6] and β-actin (Sigma) antibodies. Protein-antibody complexes were detected using horseradish peroxidase-linked secondary anti-mouse or anti-rabbit (Amersham Biosciences), and the signal was developed using enhanced chemiluminescence (Pierce) according to manufacturer's instructions. Bands were quantitated using FluorChem 8000 (Alpha Inotech Corp.) and averaged the percentages of SAFB1 expression in all analyzed tissues of SAFB1^+/- ^mice with respect to the SAFB1^+/+ ^mice

### Tumorigenesis study

For DMBA induced tumorigenesis, 8 week old virgin female BALB/c mice were treated with 1 mg/week DMBA (Sigma-Aldrich Inc, St. Louis, MO), dissolved in cotton seed oil (Sigma-Aldrich Inc, St. Louis, MO) by gavage for 6 consecutive weeks. Control mice received cotton seed oil only.

After weaning (Wnt tumor), or one week after DMBA treatment (DMBA tumor) mice were monitored 3 times/week for tumor formation by visual inspection and palpation. Tumors were measured with a calliper and volume was calculated according to the formula (W*W*L)/2, where W is the width and L is the length. Mice where euthanized when tumor volume reached > 1000 mm^3 ^or general health conditions deteriorated due to unknown reasons. All animals that did not develop palpable tumors were harvested at the end of the study period (50–52 weeks after DMBA treatment). Necropsies were performed to determine incidence of non-palpable tumors or blood disorders. Mice were injected with 100 mg/kg BrdU 2 hr prior to sacrifice for subsequent measurements of proliferation. At the time of sacrifice, tumors and lungs were removed. Part of the tumors was used for paraffin-embedding and the remaining part was frozen at -80°C. Blood samples were obtained from some of the animals and analyzed for hematological parameters. Control mice without the Wnt-1 transgene of both groups SAFB1^+/- ^female and male mice were monitored for up to 18 months. In the Wnt-1 study, both breeding and non-breeding males and only virgin females were used for data collection.

### Histological analysis

Lung and tumor cross-sections were fixed in fresh 4% paraformaldehyde overnight, and stored in 70% ethanol until embedding. Samples were paraffin embedded, sectioned, and H&E stained using standard protocols, by the Breast Center Pathology Core at BCM. For the immunohistochemical (IHC) staining, we have used rabbit anti-caspase 3 antibody (Cell Signalling) to measure apoptosis and BrdU In Situ detection kit II (BD Pharmingen) to analyze proliferation. In the Wnt study we have additionally stained with anti-phosphorylated H3 (Upstate) to measure mitotic index of the tumors.

Three photographs from different areas of the tumors were taken per each slide and the percentage of these apoptotic and proliferations marker-positive cells was calculated. Blood samples were taken from 17 DMBA treated mice (7 SAFB1^+/+^, 8 SAFB1^+/- ^and 2 control) by cardiac puncture. Haematological analyses were carried out on the Advia 120 (Bayer Diagnostics) automated hematology analyzer, and blood smears were evaluated for abnormalities.

### Statistical Analysis

For all statistical tests, p ≤ 0.05 was considered statistically significant. The time from birth (Wnt-1 tumors) or from the first DMBA treatment to the time of tumor appearance and death were subjected to Kaplan-Meier analysis.

## Abbreviations

The abbreviation used are: DMBA: 7,12-dimethylbenz(a)anthracene; BrdU: Bromodeoxyuridine; ERα: Estrogen Receptor α; H&E: Hematoxylin and Eosin; LOH: Loss of Heterozygosity; IGF-I: Insulin-Like Growth Factor-I; MMTV: Mouse Mammary Tumor Virus; OS: Overall Survival; PAH: Polycyclic Aromatic Hydrocarbon; SAFB: Scaffold Attachment Factor B; SEM: Standard Error of the Mean.

## Competing interests

The authors declare that they have no competing interests.

## Authors' contributions

BK planned and performed DMBA study and data analysis, and prepared the draft version of the manuscript. KD planned and performed Wnt-1 study, data analysis, and prepared the draft version of the manuscript. OB performed the animal experiments, AL, YL, ML and DM participated in the study design and in the scientific discussion. SO participated in the conception, supervision, and coordination of the study, and manuscript preparation. All authors have read and approved the final version of the manuscript.
